# Regulation of stomatal opening and histone modification by photoperiod in *Arabidopsis thaliana*

**DOI:** 10.1038/s41598-019-46440-0

**Published:** 2019-07-22

**Authors:** Saya Aoki, Shigeo Toh, Norihito Nakamichi, Yuki Hayashi, Yin Wang, Takamasa Suzuki, Hiroyuki Tsuji, Toshinori Kinoshita

**Affiliations:** 1 Division of Biological Science, Graduate School of Science, Nagoya University, Chikusa, Nagoya 464-8602 Japan; 2 Institute of Transformative Bio-Molecules (WPI-ITbM), Nagoya University, Chikusa, Nagoya 464-8602 Japan; 3 Institute for Advanced Research, Nagoya University, Chikusa, Nagoya 464-8602 Japan; 4 Department of Biological Chemistry, College of Bioscience and Biotechnology, Chubu University, 1200 Matsumoto-cho, Kasugai, Aichi 487-8501 Japan; 5 Kihara Institute for Biological Research, Yokohama City University, 641-12 Maioka, Totsuka, Yokohama 244-0813 Japan; 6 Present Address: Ministry of Education, Culture, Sports, Science and Technology, Chiyoda, Tokyo 100-8959 Japan; 7 Present Address: Department of Life Sciences, School of Agriculture, Meiji University, Tama, Kawasaki 214-8571 Japan

**Keywords:** Light responses, Stomata

## Abstract

Stomatal movements are regulated by many environmental signals, such as light, CO_2_, temperature, humidity, and drought. Recently, we showed that photoperiodic flowering components have positive effects on light-induced stomatal opening in *Arabidopsis thaliana*. In this study, we determined that light-induced stomatal opening and increased stomatal conductance were larger in plants grown under long-day (LD) conditions than in those grown under short-day (SD) conditions. Gene expression analyses using purified guard cell protoplasts revealed that *FT* and *SOC1* expression levels were significantly increased under LD conditions. Interestingly, the enhancement of light-induced stomatal opening and increased *SOC1* expression in guard cells due to LD conditions persisted for at least 1 week after plants were transferred to SD conditions. We then investigated histone modification using chromatin immunoprecipitation–PCR, and observed increased trimethylation of lysine 4 on histone 3 (H3K4) around *SOC1*. We also found that LD-dependent enhancement of light-induced stomatal opening and H3K4 trimethylation in *SOC1* were suppressed in the *ft-2* mutant. These results indicate that photoperiod is an important environmental cue regulating stomatal opening, and that LD conditions enhance light-induced stomatal opening and epigenetic modification (H3K4 trimethylation) around *SOC1*, a positive regulator of stomatal opening, in an *FT*-dependent manner. Thus, this study provides novel insights into stomatal responses to photoperiod.

## Introduction

Plants need stomata in the plant epidermis for gas exchange between plants and the atmosphere, providing CO_2_ uptake for photosynthesis, O_2_ efflux, and transpiration. The movements of the stomata are regulated by various environmental signals such as light, temperature, CO_2_, drought conditions and pathogens^1,2^. Among them, blue light, red light and low CO_2_ act as a positive signal for stomatal opening. Blue light activates blue light receptor phototropins and blue light signaling component-mediated activation of plasma membrane (PM) H^+^-ATPase in guard cells^3,4^. In *Arabidopsis thaliana*, 11 PM H^+^-ATPase isoforms are recognized^5^, and all genes are expressed in guard cell protoplasts^6^. Blue light activates PM H^+^-ATPase by phosphorylating the penultimate residue, threonine, and 14-3-3 protein binding to the phosphorylated C-terminus^7,8^. Next, negative electrical potential was occurred inside the PM by blue light-activated PM H^+^-ATPase and inward-rectifying K^+^ channels induced K^+^ uptake through voltage-gated in response to the negative electrical potential. Finally, water potential changes turgor and volume in guard cells, leading to stomatal opening^2,4^.

In the early phase of blue light signaling pathway, a protein kinase, *BLUE LIGHT SIGNALING1* (*BLUS1*), and type 1 protein phosphatase (PP1) have an important role between phototropins and PM H^+^-ATPase^9–11^. BLUS1 directly binds with phototropins in guard cells. Phosphorylation of BLUS1 by phototropins and kinase activity of BLUS1 are both essential for the PM H^+^-ATPase activation. PP1 is composed of both a catalytic subunit and a regulatory subunit. Both PP1 subunits may be involved in signal transduction from phototropins to PM H^+^-ATPase. Recently, BLUE LIGHT-DEPENDENT H^+^-ATPASE PHOSPHORYLATION (BHP), a Raf-like kinase, was reported as a novel signaling component for blue light-induced stomatal opening^12^. BHP interacts with BLUS1 but not with phototropins or PM H^+^-ATPase, and forms an early signaling complex with phototropins via BLUS1 in guard cells.

Furthermore, recent studies have indicated that mRNAs of photoperiodic flowering components, such as *GIGANTEA* (*GI*), *CONSTANS* (*CO*), *FLOWERING LOCUS T* (*FT*), *TWIN SISTER OF FT* (*TSF*), and *SUPPRESSOR OF OVEREXPRESSION OF CO 1* (*SOC1*), are exist in guard cells and these components positively enhance light-induced stomatal opening in *A. thaliana*^13–15^. In addition, the well characterized blue light photoreceptor *CRYPTOCHROME* (*CRY*), which regulates photoperiodic flowering, has a function in the regulation of light-induced stomatal aperture via regulation of *FT* and *TSF* expression^14^. These findings suggest that photoperiod has a substantial effect on stomatal opening. Recently, Hassidim *et al*. (2017) reported that long-day (LD) conditions induce stomatal opening in *A. thaliana* 2 h before lights-on, but that short-day (SD) conditions do not induce stomatal opening before lights-on, and that the amplitude of the stomatal aperture is smaller throughout the day under SD conditions than under LD conditions^16^. However, to date, there has been no detailed analysis of the relationship between photoperiod and light-induced stomatal opening.

*SOC1* gene encodes a multifunctional MADS box protein^17–20^ that regulates the timing of flowering, and floral pattern and meristem determinacy^21–23^. *SOC1* expression is also mediated by *FT* in Arabidopsis guard cells^14^, and *SOC1* acts as a positive regulator in light-induced stomatal opening^15^. The transcription factor MYB60 is specifically expressed in guard cells, and a null mutant of AtMYB60 exhibited reduced light-induced stomatal opening^24^.

In this study, we investigated the effect of photoperiod on light-induced stomatal opening in *A. thaliana* and found that LD conditions enhanced light-induced stomatal opening and *SOC1* expression via *FT* and increased expression level of a PM H^+^-ATPase isoform, *AHA5*, in guard cells. We also determined that the enhancement of light-induced stomatal opening and *SOC1* expression in guard cells by LD conditions persisted for at least 1 week after plants were transferred to SD conditions, and that LD conditions induced *FT*-dependent epigenetic regulation [trimethylation of lysine 4 of histone 3 (H3K4)] of *SOC1*, a downstream transcription factor of *FT*.

## Results and Discussion

**Figure 1:**
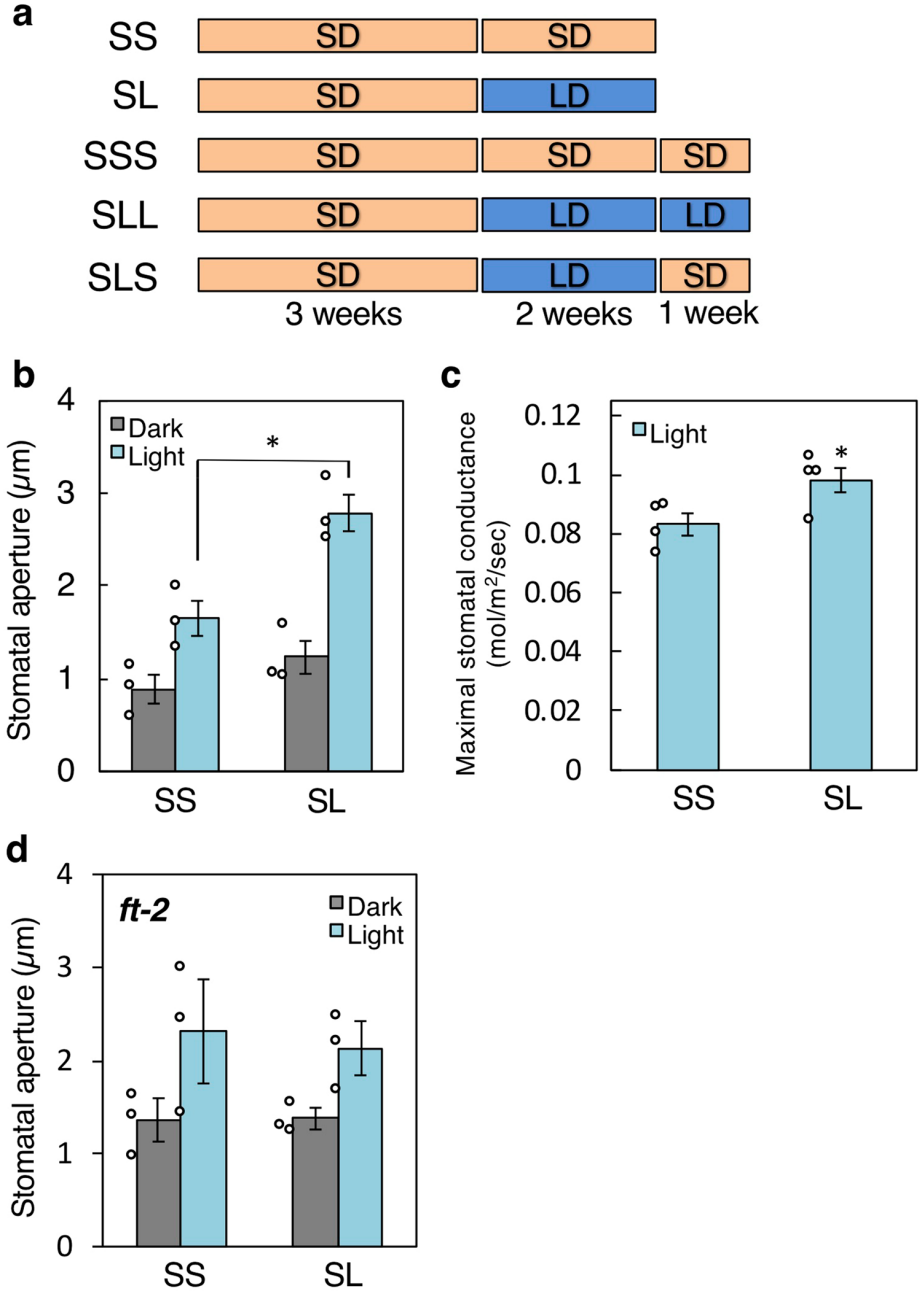
Long-day (LD) conditions enhanced light-induced stomatal opening via *FT*. (**a**) Summary of plant growth conditions. SS: short-day (SD) conditions for 5 weeks; SL: SD conditions for 3 weeks followed by LD conditions for 2 weeks; SSS: SD conditions for 6 weeks; SLL: SD conditions for 3 weeks followed by LD conditions for 3 weeks; SLS: SD conditions for 3 weeks followed by LD conditions for 2 weeks and SD conditions for 1 week. (**b**) Light-induced stomatal opening in epidermis from SS and SL plants. Stomatal apertures were determined 3 h after the start of light illumination or in darkness. Data are means of three independent experiments ± standard error (SE) (two-sided Student’s *t*-test, *P = 0.014). (**c**) Light-induced increase in stomatal conductance in intact leaves from SS and SL plants. Maximal stomatal conductance was calculated as the average conductance from 100 to 120 min after the start of light illumination. Data are means of four measurements in SS and SL plants ± SE (two-sided Student’s *t*-test, *P = 0.048). (**d**) Light-induced stomatal opening in epidermis of *ft-2* mutant plants. Data are means of three independent experiments ± SE. Circles in (**b**,**d**) indicate means of 25 stomatal apertures, with five epidermal fragments for each experiment.

To clarify the effects of photoperiod on stomatal opening in response to light, we investigated light-induced stomatal opening in plants grown under LD and SD conditions. Kinoshita *et al*. (2011) showed that plants grown under LD and SD conditions differed significantly in shape^13^; therefore, we first established the conditions for plant growth. Plants were grown under SD conditions for 3 weeks, and then transferred to separate SD conditions for 2 weeks (SS) or to LD conditions for 2 weeks (SL); both groups showed similar leaf area, suggesting that there was no significant difference in plant growth (Fig. 1a; Supplementary Fig. 1; Supplementary Table 1). We then used these plants for further experiments. Stomata in the epidermis from both SS and SL plants showed light-induced stomatal opening, with SL plants showing significantly larger stomatal aperture than SS plants in response to light (Fig. 1b). We further investigated the light-induced increase of stomatal conductance in rosette leaves from SS and SL plants with a gas exchange system (Supplementary Fig. 2), and found that maximal stomatal conductance was 18% greater in SL plants than in SS plants (Fig. 1c). Note that photosynthetic activity of SL plants is significantly higher than that of SS plants during 10 to 30 min after the start of illumination, but there is no significant difference after 30 min (Supplementary Fig. 3). Under the same conditions, we detected no significant difference in stomatal density between plant groups (Supplementary Table 1). These results indicate that photoperiod is an important environmental cue regulating stomatal opening, and that LD conditions enhance light-induced stomatal opening without affecting stomatal development. Furthermore, we found that stomata in the loss-of-function mutant of *FT*, *ft-2*^25^, did not show LD-dependent enhancement of light-induced stomatal opening (Fig. 1d), indicating that this process is mediated by *FT*.

**Figure 2:**
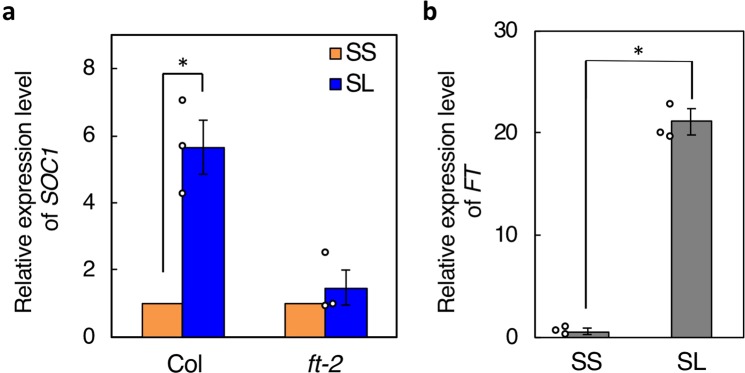
Expression of *SOC1* and *FT* (ZT16). (**a**) Quantitative reverse-transcription polymerase chain reaction (qRT-PCR) analysis of *SOC1* expression in guard cell protoplasts (GCPs) from Col and guard cell-enriched epidermal fragments from the *ft-2* mutant. Data are means of three independent experiments ± SE (two-sided Student’s *t*-test, *P = 0.004). Circles indicate individual values of three replicates. (**b**) qRT-PCR analysis of *FT* expression in GCPs from SS and SL plants. Experiments repeated on three occasions yielded similar results.

To clarify the mechanism of stomatal regulation by photoperiod, we first analyzed gene expression in guard cell protoplasts (GCPs) isolated from SS and SL plants in the evening (at zeitgeber time [ZT]16) by microarray (Supplementary Data 1). As shown in Table 1, the expression of several genes including *SOC1*, *FRUITFULL*, *ATRALF1, hypothetical protein, ATTSPO, CCOAMT, ATSUC1*, *and ATCKX5* was repeatedly increased in GCPs from SL plants, showing a greater than 2-fold increase compared with those from SS plants. Among these genes, *SOC1*, a multifunctional MADS box protein, has been reported to act as a positive regulator of light-induced stomatal opening^15^. We confirmed increased *SOC1* expression in GCPs from SL plants using quantitative reverse-transcription PCR (qRT-PCR) (Fig. 2a). In contrast, we observed no clear LD-dependent increase in *FT* expression by microarray, probably because *FT* expression levels were too low. Therefore, we conducted qRT-PCR analysis to detect *FT* expression, and found that *FT* was also significantly increased in GCPs from SL plants isolated in the evening (ZT16) (Fig. 2b). Furthermore, we found that the LD-dependent increase in *SOC1* expression was severely suppressed in guard cell-enriched epidermal fragments from the *ft-2* mutant (Fig. 2a). Together, these results suggest that *SOC1* is involved in LD-dependent enhancement of light-induced stomatal opening downstream of *FT*.

**Table 1:** Microarray analysis of guard cell protoplasts (GCPs).

AGI No.	Name	SS	SL	Fold Change (SL/SS)	*P* value
AT2G45660	*SOC1*	165 ± 12	787 ± 140	4.68 ± 0.53	0.012
AT5G60910	*FRUITFULL*	290 ± 58	763 ± 82	2.73 ± 0.24	0.009
AT1G02900	*ATRALF1*	1376 ± 241	3061 ± 396	2.33 ± 0.39	0.022
AT4G30650	*Hypothetical protein*	1871 ± 140	4155 ± 627	2.28 ± 0.43	0.024
AT2G47770	*ATTSPO*	648 ± 171	1296 ± 122	2.27 ± 0.56	0.037
AT1G67980	*CCOAMT*	328 ± 42	727 ± 129	2.18 ± 0.17	0.043
AT1G71880	*ATSUC1*	819 ± 87	1647 ± 46	2.08 ± 0.31	0.001
AT1G75450	*ATCKX5*	2805 ± 561	5465 ± 675	2.02 ± 0.21	0.039

Plants were grown under short-day (SD) conditions for 3 weeks, and then transferred to separate SD conditions for 2 weeks (SS) or to long-day (LD) conditions for 2 weeks (SL). GCPs from SS and SL plants were then used for analysis. Data are means of three independent experiments; significant differences were determined using two-sided Student’s *t*-tests, at a significance level *P* < 0.05. AGI, Arabidopsis Genome Initiative.

*SOC1* overexpression in guard cells enhances light-induced stomatal opening and increases expression levels of plasma membrane (PM) H^+^-ATPase isoforms^15^. Therefore, we analyzed the gene expression of GCPs isolated from SS and SL plants in the morning (ZT4) by RNA sequencing (RNA-seq) analysis (Supplementary Data 2), because light-induced stomatal opening is observed around ZT4 under growth conditions. The 21 genes including *SOC1* that showed a greater than 2-fold change in expression are listed in Supplementary Table 2. However, to our knowledge, these genes except for *SOC1* are not involved in stomatal opening and closing. We then analyzed the expression of genes involved in light-induced stomatal opening, which is mediated by several components including blue light receptors phototropin^3^, BLUS1^9^, BHP^12^, PP1 (TOPP and PRSL1)^10,11^, PM H^+^-ATPase^25,26^, K^+^ channels (KAT1, KAT2, and AKT1)^2,4^, and MYB60^24^ (Table 2). Interestingly, the expression levels of *BHP*, *AHA5*, and *AHA11* were repeatedly increased in GCPs from SL plants (Supplementary Fig. 4). In particular, we observed a marked increase in reads per million (RPM) of *AHA5*, which exhibited the second highest expression level among the *AHA* isoforms in the SS condition. We confirmed this finding using qRT-PCR (Supplementary Fig. 5). *AHA5* was significantly increased in the GCPs of SL plants. Given that increased expression of PM H^+^-ATPase in guard cells increases the magnitude of light-induced stomatal opening^27^, it is possible that LD-dependent enhancement of stomatal opening in SL plants is at least partly due to an increase in the expression level of *AHA5* in guard cells. Further research is needed to determine how *SOC1* induces PM H^+^-ATPase isoform expression in guard cells.

**Table 2:** Expression levels of genes related to light-induced stomatal opening.

AGI No.	Name	RPM in SS	RPM in SL	Fold Change (SL/SS)	FDR	*P* value
AT3G45780	*PHOT1*	110 ± 60.9	129 ± 50.0	1.16	1.00	0.536
AT5G58140	*PHOT2*	269 ± 130	300 ± 54.5	1.12	1.00	0.639
AT4G14480	*BLUS1*	26.5 ± 17.7	15.3 ± 3.39	0.58	1.00	0.064
AT4G18950	*BHP*	6743 ± 618	7537 ± 663	1.12	1.00	0.662
AT2G29400	*TOPP1*	54.8 ± 7.21	54.7 ± 5.86	1.00	1.00	0.977
AT5G59160	*TOPP2*	36.9 ± 9.20	37.2 ± 4.87	1.01	1.00	0.943
AT1G64040	*TOPP3*	135 ± 23.2	120 ± 13.3	0.88	1.00	0.560
AT2G39840	*TOPP4*	37.2 ± 7.15	36.0 ± 6.33	0.97	1.00	0.891
AT3G46820	*TOPP5*	18.7 ± 7.46	16.7 ± 6.15	0.89	1.00	0.685
AT5G43380	*TOPP6*	6.75 ± 5.26	3.32 ± 1.75	0.49	1.00	0.057
AT4G11240	*TOPP7*	24.8 ± 1.29	24.9 ± 0.32	1.00	1.00	0.935
AT5G27840	*TOPP8*	105 ± 18.3	96.9 ± 17.1	0.92	1.00	0.732
AT3G05580	*TOPP9*	153 ± 37.5	136 ± 22.4	0.89	1.00	0.594
AT4G40100	*PRSL1*	0 ± 0	0 ± 0	—	—	—
AT2G18960	*AHA1*	681 ± 385	592 ± 100	0.87	1.00	0.642
AT4G30190	*AHA2*	271 ± 12.3	232 ± 17.3	0.85	1.00	0.464
AT5G57350	*AHA3*	15.4 ± 10.5	16.2 ± 8.72	1.06	1.00	0.881
AT3G47950	*AHA4*	1.33 ± 0.49	1.21 ± 0.50	0.91	1.00	0.809
AT2G24520	*AHA5*	507 ± 26.0	584 ± 44.4	1.15	1.00	0.481
AT2G07560	*AHA6*	0.02 ± 0.04	0 ± 0	0	1.00	0.229
AT3G60330	*AHA7*	1.67 ± 0.37	1.39 ± 0.25	0.83	1.00	0.560
AT3G42640	*AHA8*	6.00 ± 2.96	4.61 ± 1.71	0.77	1.00	0.412
AT1G80660	*AHA9*	4.64 ± 2.77	5.05 ± 2.06	1.09	1.00	0.795
AT1G17260	*AHA10*	1.45 ± 0.30	1.60 ± 0.19	1.10	1.00	0.725
AT5G62670	*AHA11*	8.99 ± 3.65	11.1 ± 5.21	1.24	1.00	0.432
AT5G46240	*KAT1*	105 ± 79.0	74.8 ± 34.1	0.71	1.00	0.432
AT4G18290	*KAT2*	103 ± 23.5	101 ± 8.43	0.98	1.00	0.312
AT2G26650	*AKT1*	24.7 ± 5.77	20.5 ± 2.34	0.83 ± 0.08	1.00	0.382
AT1G08810	*MYB60*	177 ± 134	112 ± 51.0	0.63 ± 0.15	1.00	0.170

The actual read counts were normalized by TMM normalization and converted to reads per million (RPM). Data are means ± SD of three independent experiments from RNA sequencing analysis of GCPs isolated at ZT4. False discovery rate (FDR) and raw *p*-value (*P* value) are calculated with edgeR.

**Figure 3:**
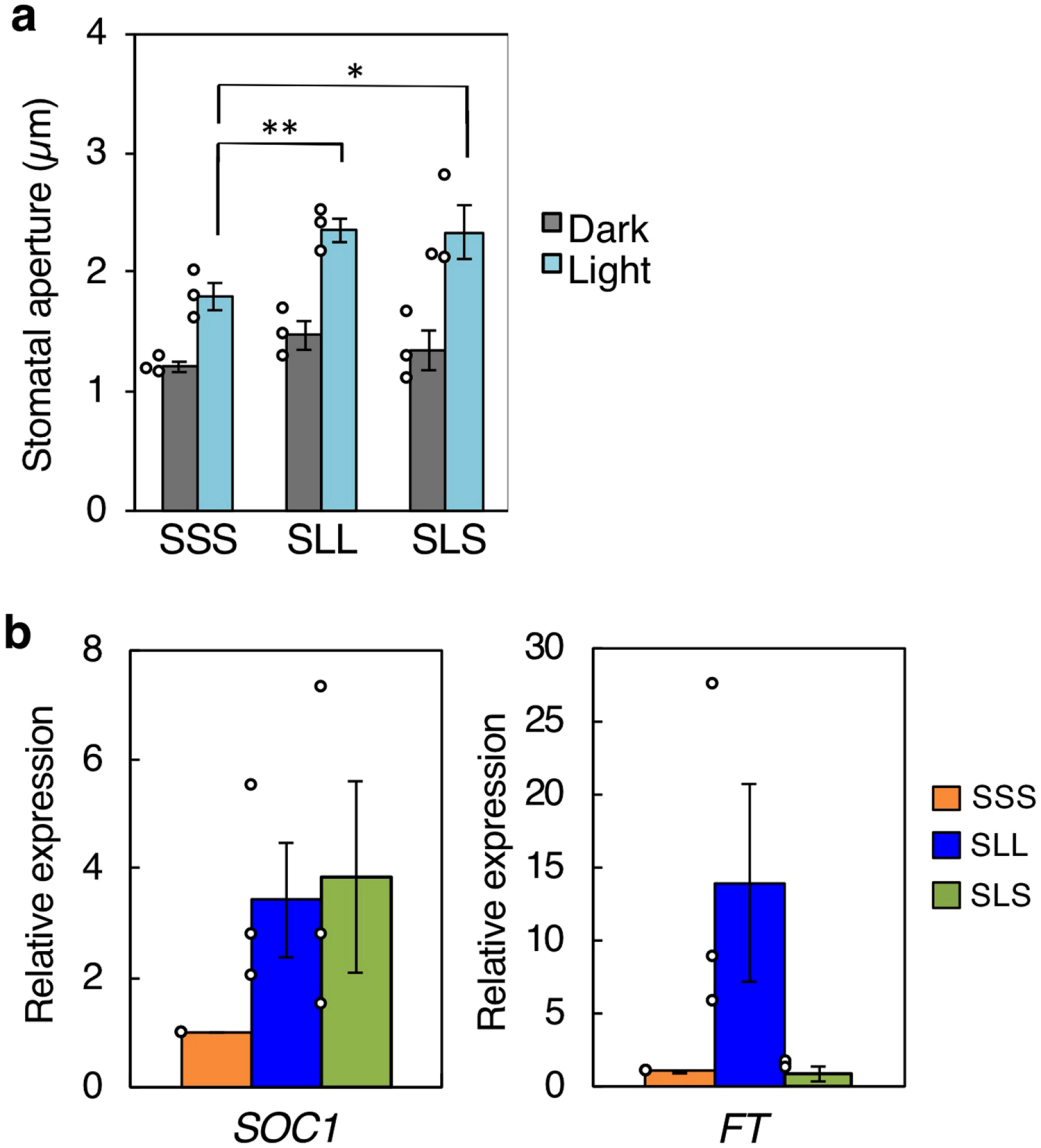
Light-induced stomatal opening and *SOC1* and *FT* expression under different conditions. (**a**) Light-induced stomatal opening in epidermis from SSS, SLL, and SLS plants. Data are means of three independent experiments ± SE (ANOVA, one-sided, *P = 0.0434, **P = 0.0400). Circles indicate means of 25 stomatal apertures from five epidermal fragments for each experiment. (**b**) qRT-PCR analysis of *SOC1* and *FT* expression in guard cell-enriched epidermal fragments from SSS, SLL, and SLS plants. Data are means of three independent experiments ± SE. Circles indicate exact values for each sample.

Next, we investigated whether LD-dependent enhancement of light-induced stomatal opening persists when the plants are returned to SD conditions. We transferred SL plants to LD conditions (SLL) or SD conditions (SLS) for 1 week (Fig. 1a). SLL plants still showed enhancement of light-induced stomatal opening. Surprisingly, SLS plants continued to exhibit enhanced light-induced stomatal opening (Fig. 3a). We then investigated *SOC1* expression levels in guard cell-enriched epidermal fragments from SSS, in which plants were grown under SD conditions for 6 weeks (Fig. 1a), SLL, and SLS plants using qRT-PCR. Consistent with the light-induced stomatal opening phenotype, *SOC1* expression was higher in both SLL and SLS plants than in SSS plants (Fig. 3b). In contrast, *FT* expression was lower in SLS plants (Fig. 3b). These results suggest that guard cells of SLS plants memorize *FT*-dependent enhancement of light-induced stomatal opening and *SOC1* expression under LD conditions for at least 1 week, even after returning to SD conditions.

**Figure 4:**
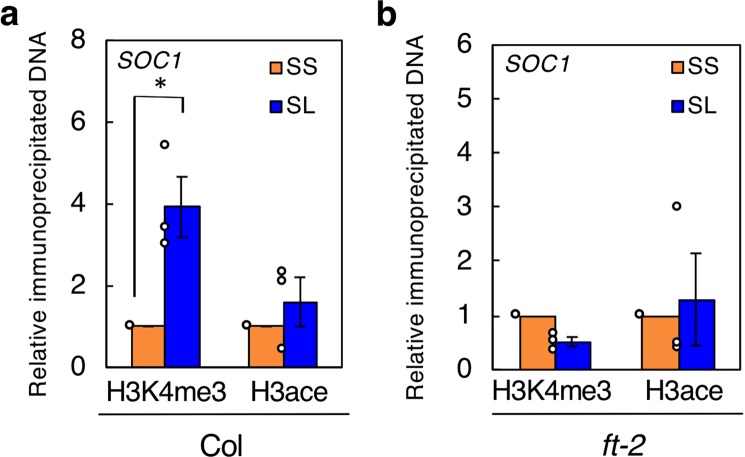
ChIP-qPCR analysis of *SOC1*. (**a**) ChIP-qPCR using GCPs from Col SS and SL plants. H3K4me3: ChIP using anti-histone H3 (trimethyl K4) antibody. H3ace: ChIP using anti-acetyl-histone H3 antibody. Data are means of three independent experiments ± SE (two-sided Student’s *t*-test, *P = 0.017). Circles indicate exact values for each sample. (**b**) ChIP-qPCR using guard cell-enriched epidermal fragments from *ft-2* SS and SL plants. Primer pairs used for ChIP-qPCR cover 3–177 bp of the *SOC1* coding region (Supplementary Table 3). Data are means of three independent experiments ± SE. Circles indicate exact values for each sample.

Epigenetic regulation is important for plant acclimation to environmental stresses and signals^28,29^, and trimethylation of lysine 4 on histone H3 (H3K4) and acetylation of lysine 9 on H3 (H3K9) are important for the upregulation of gene expressions in response to drought stress and vernalization in plants^30–32^. Therefore, we investigated the status of H3K4 trimethylation and H3K9 acetylation on *SOC1* by chromatin immunoprecipitation (ChIP)-qPCR in GCPs from Col SS and SL plants (Fig. 4a). In SL plants, H3K4 trimethylation on *SOC1* was increased; however, H3K9 acetylation was unchanged. Furthermore, the *ft-2* mutant did not exhibit LD-dependent enhancement of H3K4 trimethylation on *SOC1* in guard cell-enriched epidermal fragments from the *ft-2* mutant (Fig. 4b). These results indicate that LD conditions induce H3K4 trimethylation on *SOC1* in guard cells, and that this modification is mediated by *FT*.

Long-day plants such as *A. thaliana* initiate floral induction under LD conditions^33^. The results of the current study clearly indicate that stomata open more widely in response to light via *FT* in plants grown under LD conditions than under SD conditions (Fig. 1b–d). We previously showed that enhanced light-induced stomatal opening induces increased photosynthesis and plant growth using transgenic Arabidopsis plants overexpressing PM H^+^-ATPase in guard cells^27^. Together, these results suggest that the enhancement of light-induced stomatal opening by LD conditions may be beneficial to plants in the reproductive phase through providing much energy and nutrient supplied by increased photosynthesis and transpiration. It would be interesting to determine whether SD conditions enhance light-induced stomatal opening in short-day plants such as rice.

**Figure 5:**
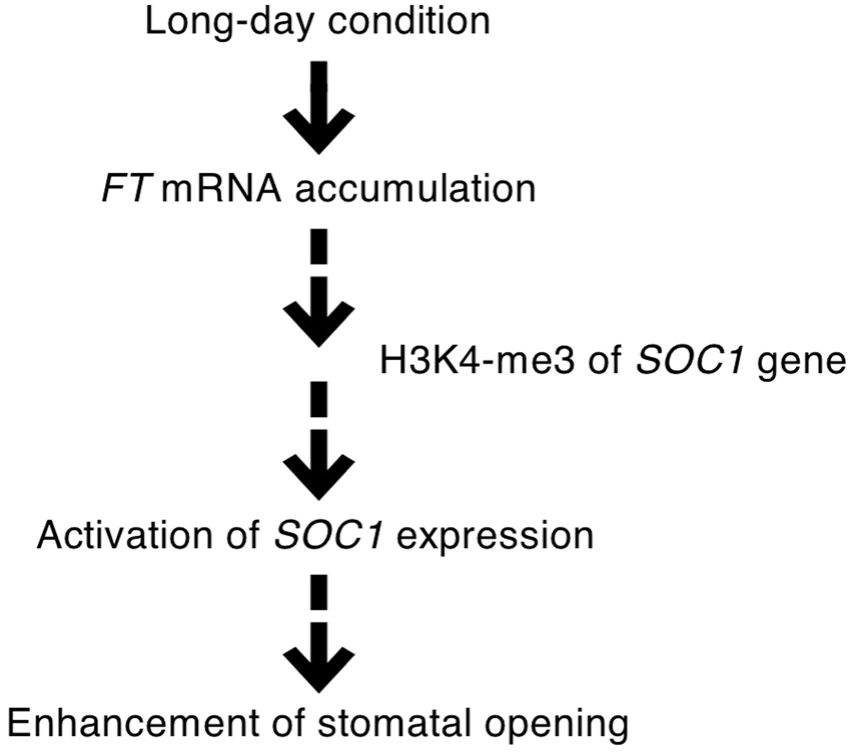
Hypothetical model of stomatal regulation under LD conditions.

The transfer of plants to LD conditions for 2 weeks enhanced light-induced stomatal opening, *FT* expression, which altered H3K4 trimethylation on *SOC1*, and *SOC1* expression in guard cells. Even when plants were transferred to SD conditions, the enhanced light-induced stomatal opening and *SOC1* expression in guard cells were irreversibly retained for at least 1 week. We call this phenomenon “LD memory” (Fig. 5). These results suggest that H3K4 trimethylation on *SOC1* in response to LD conditions is likely to lead to LD memory and enhanced *SOC1* expression. However, it remains unclear whether *FT*-dependent H3K4 trimethylation on *SOC1* is required to enhance *SOC1* expression and light-induced stomatal opening. Further study is required to clarify the relationship between H3K4 trimethylation on *SOC1* and the enhancement of light-induced stomatal opening. It has been demonstrated that temperature has a significant effect on *FT* expression, and that the bHLH transcription factor PHYTOCHROME INTERACTING FACTOR 4 (PIF4) mediates temperature-dependent *FT* expression^34,35^. Therefore, it is possible that H3K4 trimethylation on *SOC1* is an important mechanism to ensure a stable response under unstable temperature conditions.

In conclusion, we demonstrated that photoperiod is an important environmental cue that regulates stomatal opening. LD conditions enhanced light-induced stomatal opening and epigenetic modification (H3K4 trimethylation) around *SOC1*, a positive regulator of stomatal opening, via *FT* in *A. thaliana* guard cells. Our results provide novel insights for studies of stomatal physiology and photoperiodic flowering physiology in shoot apical meristem. Further research is required to clarify how *FT* induces H3K4 trimethylation on *SOC1* and the physiological significance of LD-dependent enhancement of light-induced stomatal opening and plant LD memory.

## Methods

### Plant materials and growth conditions

*A. thaliana gl1* [Columbia (Col), carrying the homozygous recessive *gl1* gene] and Col were used as the wild type. The background ecotype of *ft-2* is Col (introgressed)^36^. Arabidopsis seeds in water were incubated at 4 °C for three days and sown directly on surface of the soil. Plants were grown in soil in a growth chamber (CLE-303, TOMY) as shown in Fig. 1a. SD conditions: white light for 8 h (100 µmol m^−2^ s^−1^)/darkness for 16 h at 22–24 °C in 50–70% relative humidity. LD conditions: white light for 16 h (41 µmol m^−2^ s^−1^)/darkness for 8 h at 22–24 °C in 50–70% relative humidity. We used different light intensities for SD and LD conditions because these were the optimal conditions to obtain plants of the same size under the specified photoperiods.

### Isolation of GCPs and epidermal fragments

GCPs and guard cell-enriched epidermal fragments were isolated from mature rosette leaves as previously described^12^.

### Stomatal aperture measurements

Epidermal fragments isolated from dark-adapted plants in a basal buffer (5 mM MES-BTP [pH 6.5], 50 mM KCl, and 0.1 mM CaCl_2_) were illuminated with blue light at 10 µmol m^−2^ s^−1^ superimposed on a background red light at 50 µmol m^−2^ s^−1^ at room temperature for 3 h^3^. In each independent experiment, we measured 25 stomatal apertures in the abaxial epidermis (5 stomata per epidermal fragment) using a microscope. All data represent means of three independent experiments with standard error (SE). Light-emitting photodiodes (ISL-150 × 150-RB, CCS) were used as red and blue light sources for measurement of stomatal opening. A quantum meter LI-250 (LI-COR) was used for determination of photon flux densities.

### Gas exchange measurements

Gas-exchange measurements were performed using the LI-6400XT system (LI-COR) according to a previously described method^27^. Briefly, mature leaves from *A. thaliana* plants were clamped in a standard LI-6400 chamber and illuminated from the adaxial side with white light at 150 µmol m^−2^ s^−1^ by a fiber optic illuminator with a halogen projector lamp (15 V/150 W) (Moritex) as a light source. Flow rate, leaf temperature, relative humidity, and ambient CO_2_ concentration were kept constant at 500 µmol s^−1^, 24 °C, 30–40%, and 400 µL L^−1^, respectively. Maximal stomatal conductance was calculated as the average conductance from 100 to 120 min after the start of light illumination.

### Microarray and RNA-seq analyses

RNA samples were isolated from GCPs of SS and SL plants at ZT16 using an RNeasy Plant Mini Kit (Qiagen). Labeling for each RNA (50 µg) was carried out with LIQA or LIQA WT (Agilent), according to the supplier’s protocol. We hybridized 1.65 µg of cRNA using the Arabidopsis Oligo 44 K DNA microarray chip (ver. 4.0, Agilent) at 65 °C for 17 h. Signals were scanned and normalized using the Features extraction software (Agilent). Data normalization was performed using the *limma* package in R software^37^.

For RNA-seq analysis, total RNA was extracted from GCPs collected from SS and SL plants at ZT4 using a TRIzol Plus RNA Purification Kit (Thermo Fisher Scientific). Complementary DNA libraries were constructed using a TruSeq RNA Sample Prep Kit v. 2 (Illumina) and sequenced using a NextSeq 500 system (Illumina). Base calling of sequence reads was performed using the NextSeq 500 pipeline software. Only high quality sequence reads (50 continuous nucleotides with quality values > 25) were used for mapping. Reads were mapped to Arabidopsis TAIR10 transcripts using Bowtie software^38^. Experiments were repeated three times separately. We obtained 12.6–16.9 million sequence reads per experiment. Normalization of read counts and statistical analysis were performed using the EdgeR package^39,40^. EdgeR was conducted by web tool Degust Ver. 3.1.0 (http://degust.erc.monash.edu). Obtained RPM values were further analyzed using Excel. To find up-regulated genes, low expression genes (cut-off: RPM < 3) were excluded and the genes that showed a greater than 2-fold change were filtered (Supplementary Table 2).

### qRT-PCR analysis

Total RNAs extracted from GCPs and guard cell-enriched epidermal fragments were used for RT-PCR analysis as previously described^13,14^ using the primer pairs listed in Supplementary Table 3.

### ChIP-qPCR analysis

ChIP was performed using anti-histone H3 (tri methyl K4) antibody (Abcam) and anti-acetyl-histone H3 antibody (Millipore). The amount of immunoprecipitated chromatin was determined by qPCR analysis as previously described^41^. The primer pairs used for ChIP-qPCR covered 3–177 bp of the *SOC1* coding region (Supplementary Table 3).

## Supplementary information

Supplementary information

Dataset 1

Dataset2

## Data Availability

RNAseq data that support the findings of this work have been deposited in the DNA Data Bank of Japan (DDBJ) under accession number DRA006227. Microarray data have been deposited in the Gene Expression Omnibus under accession number GSE104436.
