# Supplementary material for: Regulation of stomatal opening and histone modification by photoperiod in Arabidopsis thaliana
Source: Sci Rep. 2019 Jul 22;9:10054. doi: 10.1038/s41598-019-46440-0 (PMC6646381; doi:10.1038/s41598-019-46440-0)
Supplement: Supplementary file 1 — Supplementary information [file 41598_2019_46440_MOESM1_ESM.pdf]

Supplementary Information

Regulation of stomatal opening and histone modification by photoperiod in *Arabidopsis thaliana*

Saya Aoki[1,6]†, Shigeo Toh[1,7]†, Norihito Nakamichi[2], Yuki Hayashi[1], Yin Wang[3], Takamasa Suzuki[4], Hiroyuki Tsuji[5], Toshinori Kinoshita[1,2]*

[1]Division of Biological Science, Graduate School of Science, Nagoya University, Chikusa, Nagoya 464-8602, Japan
[2]Institute of Transformative Bio-Molecules (WPI-ITbM), Nagoya University, Chikusa, Nagoya 464-8602, Japan
[3]Institute for Advanced Research, Nagoya University, Chikusa, Nagoya 464-8602, Japan
[4]Department of Biological Chemistry, College of Bioscience and Biotechnology, Chubu University, 1200 Matsumoto-cho, Kasugai, Aichi 487-8501, Japan
[5]Kihara Institute for Biological Research, Yokohama City University, 641-12 Maioka, Totsuka, Yokohama 244-0813, Japan
[6]Present address: Ministry of Education, Culture, Sports, Science and Technology, Chiyoda, Tokyo, 100-8959, Japan
[7]Present address: Department of Life Sciences, School of Agriculture, Meiji University, Tama, Kawasaki, 214-8571, Japan
†These authors contributed equally to this work.

*Corresponding author: Toshinori Kinoshita
Institute of Transformative Bio-Molecules (WPI-ITbM), Nagoya University, Chikusa, Nagoya 464-8602, Japan
Tel/ Fax: +81-52-789-4778
E-mail: kinoshita@bio.nagoya-u.ac.jp

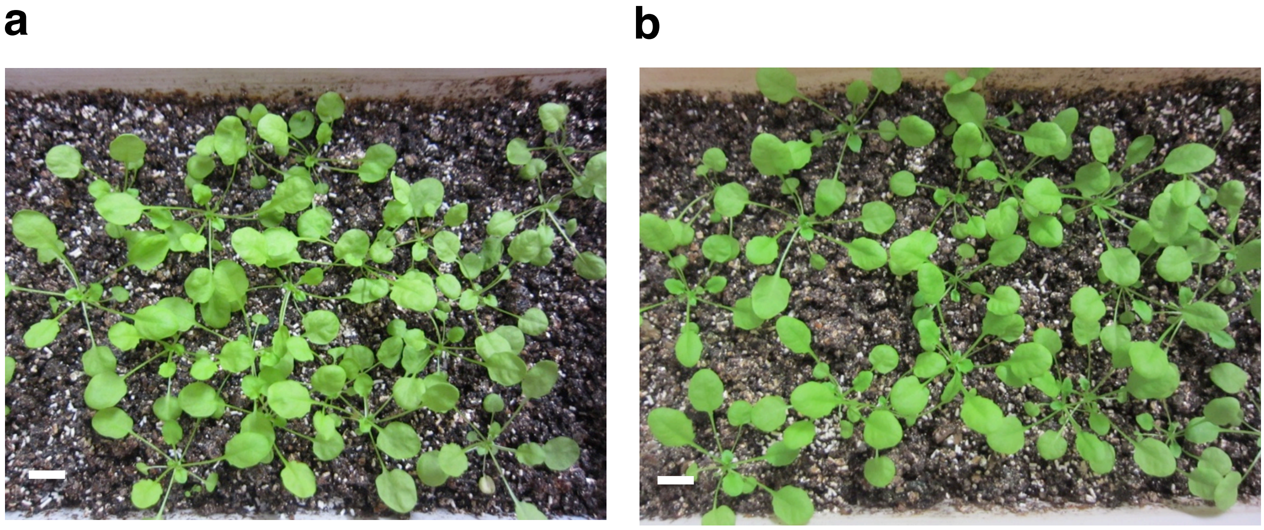

Supplementary Figure 1. Plant phenotypes grown under different conditions. a) Plants were grown under short-day (SD) conditions for 3 weeks, and then transferred to SD conditions for 2 weeks (SS). **b)** Plants were grown under SD conditions for 3 weeks, and then transferred to long-day (LD) conditions for 2 weeks (SL). Bars indicate 1 cm.

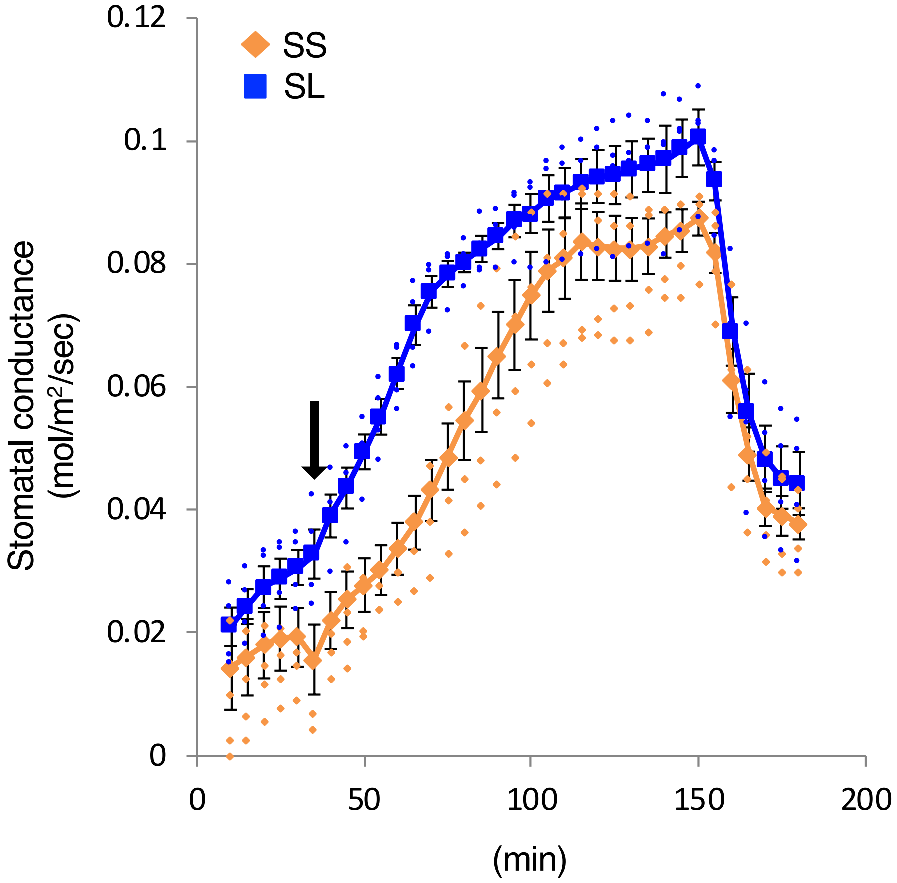

Supplementary Figure 2. Time course of light-induced increase of stomatal conductance in SS and SL plants. Data are means of four measurements in SS and SL plants ± SE. Fig. 1c was obtained from this figure. Arrow indicates start of light illumination. Circles indicate exact values for each sample.

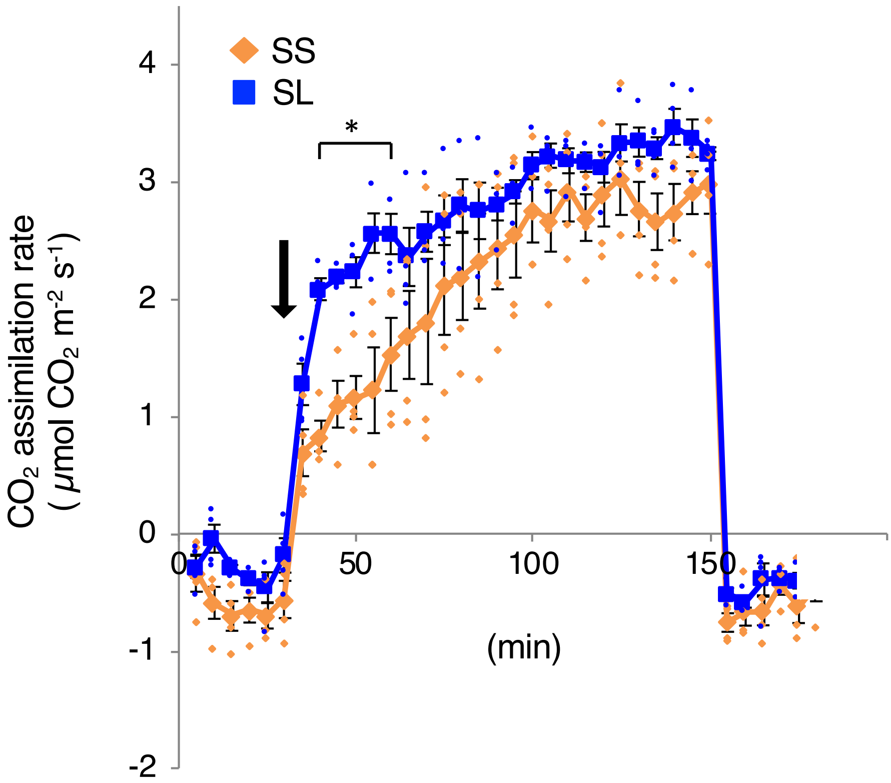

Supplementary Figure 3. Photosynthetic activity in SS and SL plants. Time course of CO_2 assimilation in SS and SL plants. Data are means of four measurements in SS and SL plants ± SE (two-sided Student's *t*-test, $^*P < 0.05$). Arrow indicates start of light illumination. Circles indicate exact values for each sample.

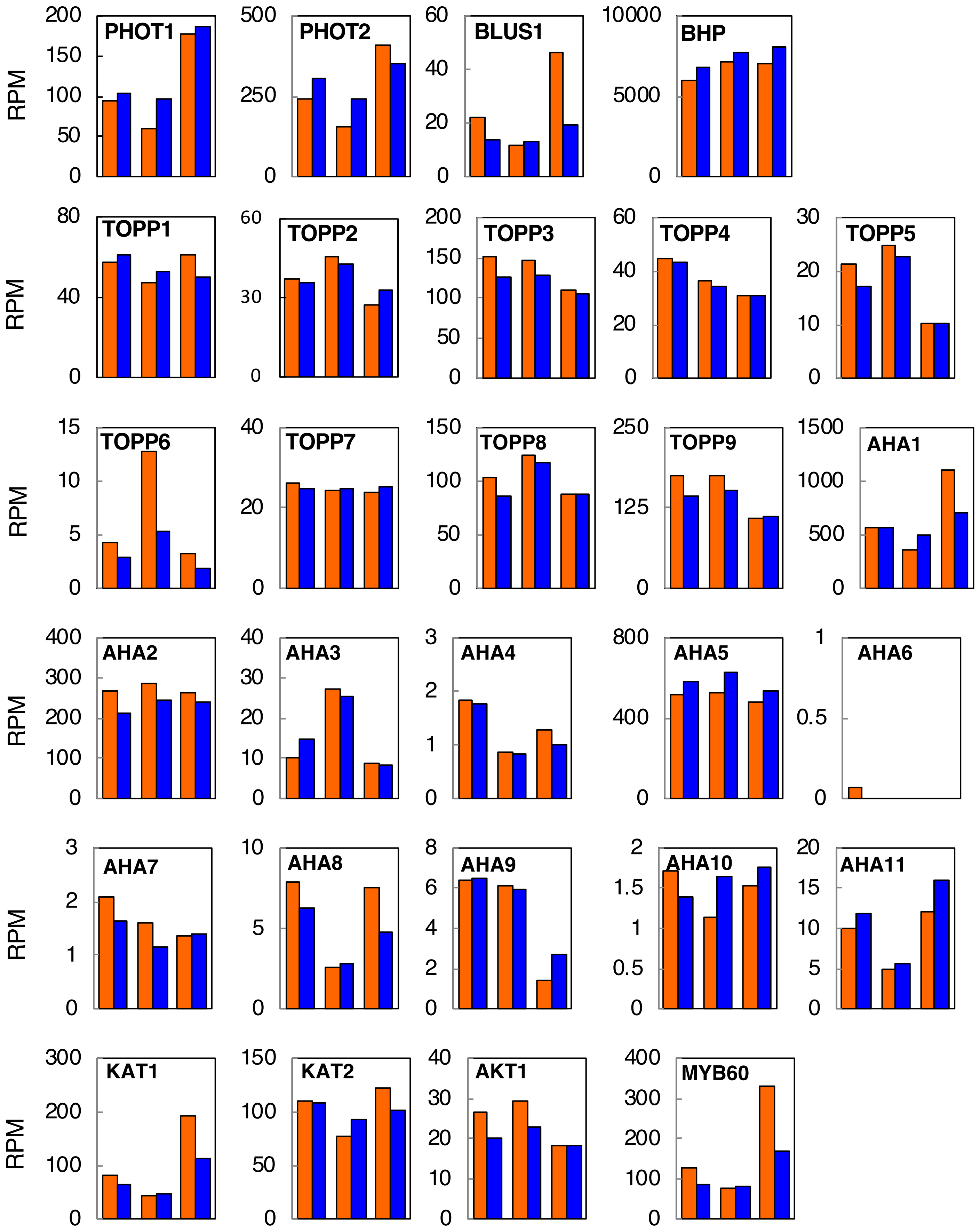

Supplementary Figure 4. Individual RPM values of genes listed in Table 2. The bar graphs show the individual RPM values of three biological replicates that are used for means listed in Table 2.

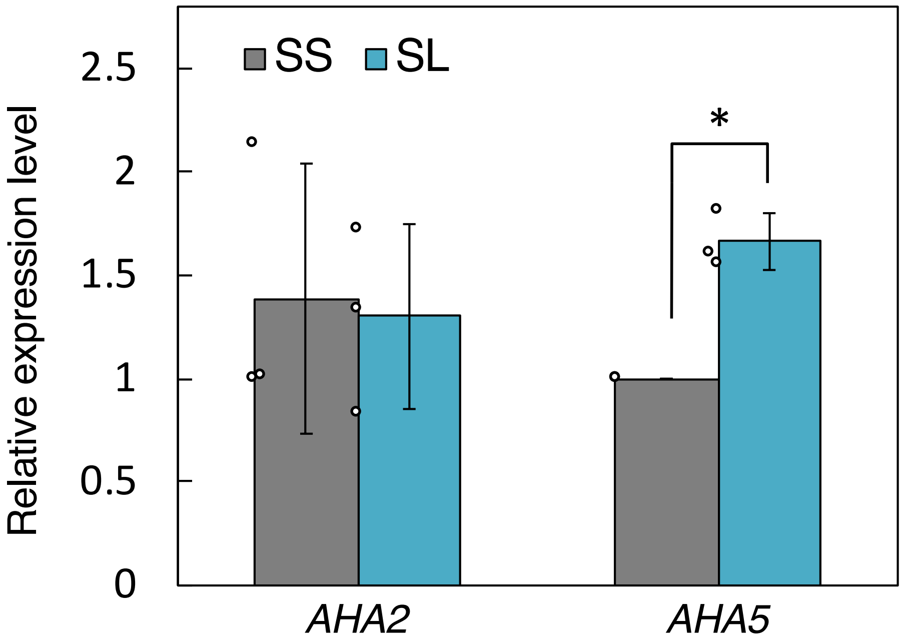

Supplementary Figure 5. Expression levels of plasma membrane H^+-ATPase isoforms (*AHA2* and *AHA5*). Quantitative reverse-transcription polymerase chain reaction (qRT-PCR) analysis of *AHA2* and *AHA5* expression in guard cell protoplasts (GCPs) collected at ZT4 from SS and SL plants. Data are means of three independent experiments ± SD. Circles indicate exact values for each sample. Asterisks indicate statistical significance (two-sided Student's *t*-test, $*P = 0.001$).

Supplementary Table 1. Stomatal density and leaf area in SS and SL plants

	Density (number of stomata per mm^2)	SD	Leaf area (cm^2)	SD
SS	91.9	± 9.4	1.5	± 0.35
SL	100.5	± 7.2	1.4	± 0.31

Plants were grown under short-day (SD) conditions for 3 weeks, and then transferred to separate SD conditions for 2 weeks (SS) or to long-day (LD) conditions for 2 weeks (SL). Stomatal density was counted from replicas of abaxial epidermis from mature leaves using varnish. Date represent means of densities in 5 different positions with SD (Student's t test, two-sided, $P = 0.13$). Leaf area was measured from scan image. Date represent means of leaf area in 18 individual plants (Student's t test, two-sided, $P = 0.38$).

Supplementary Table 2. Up-regulated genes in RNAseq analysis of GCPs (ZT4)

AGI No.	Name	RPM in SS	RPM in SL	Fold Change (SL/SS)	FDR	*P* value
AT1G01060	*LHY*	13.7 ± 11.3	63.5 ± 45.5	4.64	0.320	0.000
AT3G09600	*RVE8*	12.1 ± 7.97	43.9 ± 20.2	3.63	0.320	0.000
AT5G60910	*FUL*	4.62 ± 1.36	13.0 ± 2.95	2.82	0.320	0.000
AT1G32900	*GBSS1*	3.11 ± 1.27	7.38 ± 7.03	2.37	1.00	0.028
AT1G53560	*Ribosomal protein*	5.61 ± 0.31	11.4 ± 2.71	2.04	1.00	0.001
AT1G64500	*THRUMIN1*	6.73 ± 5.82	15.8 ± 8.16	2.34	1.00	0.019
AT1G73190	*TIP3;1*	4.24± 2.31	9.46 ± 5.72	2.23	1.00	0.014
AT2G45660	*SOC1*	8.33 ± 7.93	23.0 ± 14.3	2.76	1.00	0.010
AT2G46830	*CCA1*	80.3 ± 74.9	194 ± 145	2.42	1.00	0.027
AT3G15354	*SPA3*	26.3 ± 20.0	54.4 ± 23.9	2.07	1.00	0.022
AT3G22840	*ELIP1*	26.6 ± 28.3	95.1 ± 115	3.58	1.00	0.016
AT3G26790	*FUS3*	8.93 ± 1.69	21.8 ± 10.1	2.44	1.00	0.000
AT3G54500	*LNK2*	160 ± 81.4	390 ± 121	2.43	1.00	0.001
AT4G08950	*EXORDIUM*	58.5 ± 16.7	121 ± 50.8	2.06	1.00	0.002
AT4G10250	*ATHSP22.0*	16.5 ± 7.87	33.8 ± 29.9	2.05	1.00	0.041
AT4G15430	*Unknown*	9.14 ± 7.86	24.4 ± 19.6	2.67	1.00	0.014
AT4G38960	*BBX19*	8.31 ± 5.31	18.7 ± 9.98	2.25	1.00	0.014
AT5G15950	*SAMDC2*	8.53 ± 1.50	19.9 ± 8.99	2.33	1.00	0.001
AT5G18670	*BAM9*	10.8 ± 3.18	23.0 ± 12.7	2.14	1.00	0.007
AT5G37260	*RVE2*	26.0 ± 6.58	54.2 ± 11.6	2.09	1.00	0.001
AT5G42760	*Unknown*	3.12 ± 2.80	10.9 ± 7.50	3.49	1.00	0.002

GCPs from SS and SL plants were used for analysis. The actual read counts were normalized by TMM normalization and converted to reads per million (RPM). Data represent means of three independent experiments with SD. False discovery rate (FDR) and raw *p*-value (*P* value) are calculated with edgeR.

Supplementary Table 3. List of primers

qRT-PCR		
SOC1	Fw	CGAGAAGCTCTCTGAAAAGTGGGG
SOC1	Rv	GGGCTACTCTCTTCATCACCTCTTCC
FT	Fw	GAACTTCTATACTTTGGTTATGGTGGATC
FT	Rv	CACAATCTCATTGCCAAAGGTTG
AHA2	Fw	GCTTTGACTTACATTGACGGCAG
AHA2	Rv	CAACAAATTCCCATGGCG
AHA5	Fw	CATAGCTCAGCTGGTGGCG
AHA5	Rv	TCCACTCAAAACATAGCGGATTC
TUB2	Fw	GCGAGTTGCGGTAGATTCGT
TUB2	Rv	TCCCAGGCTCCAAATCCA
ChIP-qPCR		
SOC1	Fw	GTGAGGGGCAAAACTCAGATG
SOC1	Rv	GCTGGCGAATTCATAAAGTTTGCC
